# Establishing and integrating a transoral robotic surgery programme into routine oncological management of head and neck cancer – a UK perspective – ERRATUM

**DOI:** 10.1017/S0022215122002249

**Published:** 2023-01

**Authors:** A Arora, A Brunet, G Oikonomou, C Tornari, J Faulkner, J Jeyarajah, P Touska, A Sandison, A Rovira, R Simo, J-P Jeannon

**Affiliations:** 1 Department of Otorhinolaryngology, Head and Neck Surgery, Guy's and St Thomas’ Hospitals NHS Trust, London, UK; 2 School of Biomedical Engineering and Imaging Sciences, King's College London, London, UK; 3 Department of Otorhinolaryngology, Head and Neck Surgery, Hospital Universitari de Bellvitge, Hospitalet de Llobregat, Barcelona, Spain; 4 Bellvitge Biomedical Research Institute (‘IDIBELL’), Hospitalet de Llobregat, Barcelona, Spain; 5 Department of Otorhinolaryngology, Head and Neck Surgery, St George's University Hospitals NHS Foundation Trust, London, UK; 6 Department of Anaesthesia, Guy's and St Thomas’ Hospitals NHS Trust London, UK; 7 Department of Radiology, Guy's and St Thomas’ Hospitals NHS Trust, London, UK; 8 Department of Histopathology, Guy's and St Thomas’ Hospitals NHS Trust, London, UK

The original version of this article was published with an incomplete affiliation list. The complete list of affiliations is shown below.

^1^Department of Otorhinolaryngology, Head and Neck Surgery, Guy's and St Thomas’ Hospitals NHS Trust, London, UK, ^2^School of Biomedical Engineering and Imaging Sciences, King's College London, London, UK, ^3^Department of Otorhinolaryngology, Head and Neck Surgery, Hospital Universitari de Bellvitge, Hospitalet de Llobregat, Barcelona, Spain, ^4^Bellvitge Biomedical Research Institute (‘IDIBELL’), Hospitalet de Llobregat, Barcelona, Spain, ^5^Department of Otorhinolaryngology, Head and Neck Surgery, St George's University Hospitals NHS Foundation Trust, London, UK, ^6^Department of Anaesthesia, Guy's and St Thomas’ Hospitals NHS Trust London, UK, ^7^Department of Radiology, Guy's and St Thomas’ Hospitals NHS Trust, London, UK and ^8^Department of Histopathology, Guy's and St Thomas’ Hospitals NHS Trust, London, UK.

The original publication has now been updated.
